# Amentoflavone protects against cisplatin-induced acute kidney injury by modulating Nrf2-mediated oxidative stress and ferroptosis and partially by activating Nrf2-dependent PANoptosis

**DOI:** 10.3389/fphar.2025.1508047

**Published:** 2025-03-05

**Authors:** Yan Zhang, Jianqiang Hu, Yanmin Zhang, Xinxin Ci

**Affiliations:** ^1^ Institute of Translational Medicine, The First Hospital of Jilin University, Jilin University, Changchun, Jilin, China; ^2^ Jilin Provincial Key Laboratory of Women’s Reproductive Health, Changchun, Jilin, China

**Keywords:** amentoflavone, CI-AKI, Nrf2, ferroptosis, PANoptosis, oxidative stress

## Abstract

**Background:**

Cisplatin is a widely used drug for the treatment of solid organ cancer, but its renal toxicity cannot be ignored. Amentoflavone (AME), a natural flavonoid compound, has remarkable pharmacological effects, including anti-inflammatory and antioxidative effects. The effect and mechanism of AME on cisplatin-induced acute kidney injury (CI-AKI) remain unclear.

**Methods:**

We investigated the effect of AME on CI-AKI using the HK-2 cell line and C57BL/6 mice. Renal function, tissue damage, and molecular markers were assessed to explore the effects of AME on oxidative stress and cell death pathways.

**Results:**

*In vitro*, AME significantly suppressed the cytotoxic effects of cisplatin on HK-2 cells. Furthermore, AME significantly inhibited cisplatin-induced ferroptosis and PANoptosis (apoptosis, pyroptosis and necroptosis). In mice with acute kidney injury induced by a single intraperitoneal injection of cisplatin, the daily administration of AME during AKI effectively improved renal function and alleviated renal tubular injury, characterized by the normalization of blood urea nitrogen (BUN) and serum creatinine (SCr) levels; it also inhibited cisplatin-induced renal ferroptosis and PANoptosis. AME is a natural antioxidant that activates the Nrf2 antioxidant pathway both *in vivo* and *in vitro*. In Nrf2 knockout mice and knockdown cells, the protective effect of AME against cisplatin-induced nephrotoxicity disappeared. However, after Nrf2 knockout, the effect of AME on ferroptosis completely disappeared, and that on PANoptosis partially disappeared.

**Conclusion:**

Amentoflavone has a protective effect on cisplatin-induced acute kidney injury via a mechanism related to the Nrf2-dependent antioxidant pathway and the regulation of ferroptosis and PANoptosis.

## 1 Introduction

The incidence of mortality caused by acute kidney injury (AKI) is greater than that caused by breast cancer, diabetes, or heart failure globally; this mortality rate has remained high for the past 50 years (Pickkers et al., 2021). AKI occurs in 30% of cancer patients treated with cisplatin, which is a major limitation of cisplatin-based cancer treatment. Because the exact molecular basis of CI-AKI is still unidentified, strategies for preventing and treating CI-AKI are limited. Currently, there is no reliable pharmacological intervention for cisplatin-induced nephrotoxicity.

The development of CI-AKI involves inflammation, mitochondrial dysfunction, oxidative stress, ER stress and cell cycle dysregulation, leading to cell death of the renal tubular epithelium and resulting in a sudden decline in kidney function (Maekawa et al., 2019; Zhu et al., 2020; Fang et al., 2021). In the field of programmed cell death (PCD) the most comprehensively studied types include apoptosis, necroptosis, pyroptosis, ferroptosis and autophagy, i.e., the main means of coordinating the elimination of diseased cells. Apoptosis, the primary mode of cell death, is a caspase-based, noninflammatory programmed cell death mechanism characterized by small apoptotic cells. Pyroptosis is mediated by inflammasomes, which form caspase-1-mediated channels in the cell membrane and lyse cells, releasing inflammatory contents. Necroptosis is associated with a variety of cytokines, is characterized by cell swelling and membrane disruption, and is mainly mediated by RIP3. Recent studies have indicated that PANoptosis involves concurrent apoptosis, pyroptosis, and necroptosis within a single group of cells (Zhu et al., 2023). Ferroptosis represents a novel form of PCD distinct from apoptosis, pyroptosis, and necroptosis and is characterized by the degradation of phospholipids and iron-dependent oxidative damage to the membrane. PCD is a complex network of interconnected interactions in which different modes of cell death do not work in isolation but rather in concert, with compensation and coordination between various pathways (Bedoui et al., 2020).

Nrf2 (Pan et al., 2023) was initially recognized as a key regulator that plays a critical role in maintaining the homeostasis of cellular redox processes and facilitating the detoxification of externally harmful substances. Nrf2 has been shown to regulate genes involved in proteostasis (Pajares et al., 2017), the pentose phosphate pathway (Tan et al., 2024), and amino acid and carbohydrate metabolism (Pang et al., 2014; Bollong et al., 2018). Oxidative stress occurs when the body is exposed to external stimuli or internal environmental perturbations. Nrf2 regulates oxidative stress by activating the downstream enzymes NAD(P)H quinone oxidoreductase (NQO1) and heme oxygenase (HO-1). Previous investigations have shown that several drugs can block apoptosis (Dang et al., 2020; Zhou et al., 2020; Zhang W. et al., 2023), pyroptosis (Lin et al., 2020; Wang et al., 2023), necroptosis (Zhan et al., 2022; Zhao et al., 2022) and ferroptosis (Cui et al., 2021; Anandhan et al., 2023) by upregulating Nrf2 expression. Considering the role of oxidative metabolic disorders in cisplatin nephrotoxicity, Nrf2 activators may provide protection against the harmful effects of cisplatin.

Amentoflavone (AME), a naturally occurring flavonoid compound initially discovered in the medicinal plant *Selaginella doederleinii* Hieron, has been successively extracted from over 120 different plant species (Xiong et al., 2021; Jian et al., 2024). It has been shown to activate Nrf2 and exhibits a wide range of beneficial biological effects, including redox regulation, anti-inflammatory, anti-apoptotic, and anti-pyroptotic effects (Ijaz et al., 2024). Accumulating evidence supports its potential as a drug candidate for a variety of diseases, such as ulcerative colitis, and its protective effect against the cardiotoxicity of the chemotherapeutic drug doxorubicin. However, the protective effects of AME against renal toxicity and the fundamental processes involved remain unexplored. In this study, we focused on the protective effects of AME against cisplatin-associated oxidative damage, ferroptosis and PANoptosis in acute kidney injury. In addition, we investigated whether Nrf2 activated by AME could mediate the inhibition of the ferroptosis and PANoptosis pathways.

## 2 Materials and methods

### 2.1 Reagents

Amentoflavone (chemical registry number 1617–53-4) was obtained from Chengdu Pufei De Biotech in Chengdu, China, with a purity of over 98%. Cisplatin (chemical registry number 15663–27-1) was purchased from MCE (Monmouth Junction, New Jersey, United States). Antibodies, including anti-Bax (catalog number 2772 S), anti-BCL-2 (catalog number 3498 S), and anti-Cleaved Caspase-3 (catalog number 9661 S) were provided by Cell Signaling Technology (Massachusetts, United States). Anti-GSDMD, anti-ASC, anti-Caspase-1, and anti-RIP3 antibodies were purchased from ZEN-BIOSCIENCE (Chengdu, China). The anti-MLKL antibody was provided by ABclonal Technology (Wuhan, China). Anti-β-actin (catalog number ab252556), anti-Nrf2 (catalog number ab62352), anti-NQO1 (catalog number ab80588), anti-HO-1 (catalog number ab52947), and anti-GCLC (catalog number ab207777) antibodies were supplied by Abcam (Cambridge, MA, United States). Anti-KIM-1 (catalog number AF 1817) and anti-NGAL (catalog number AF 1857) antibodies were obtained from R&D Systems (Minnesota, United States). A BCA protein quantification kit (catalog number A55860) was used to measure protein concentrations. DMEM/F12 cell culture medium (catalog number ZQ-600), Penicillin-Streptomycin Solution (catalog number CSP006), and Trypsin/EDTA solution (catalog number CSP045) were obtained from Shanghai Zhong Qiao Xin Zhou Biotechnology (Shanghai, China). Dimethyl sulfoxide (DMSO; chemical registry number 67–68-5) was obtained from Sigma Chemical Co. (St. Louis, Missouri, United States). DCFH-DA (catalog number S0033 S) was obtained from Beyotime Biotechnology (Shanghai, China). Assay kits for BUN (catalog number C013-2–1), SCr (catalog number C011-2–1), MDA (catalog number A003-1–2), MPO (catalog number A044-1–1), GSH (catalog number A006-2–1), and SOD (catalogue number A001-3–2) were obtained from Nanjing Jiancheng Bioengineering Institute in Nanjing, China.

### 2.2 Cell treatment

Human Kidney-2 (HK-2) cells were purchased from the Chinese Cell Bank (Beijing, China). HK-2 cells were cultured in DMEM/F12 basal medium supplemented with 10% fetal bovine serum, 100 U/mL penicillin and 100 U/mL streptomycin in a cell culture incubator at 37°C in 95% air and 5% carbon dioxide.

### 2.3 Cell viability

HK-2 cells were seeded in 96-well plates at a density of 1× 10^4^ cells/well. After treating with AME and cisplatin for 24 h, 10% CCK8 reagent was mixed with FBS-free DMEM/F12 medium, and 100 μL of the mixture was added to each well, after which the plate was placed in a cell culture incubator at 37°C for 1 h. The OD value for each well was measured at 450 nm in an enzyme-labelling instrument (Bio-Tek H1MFD, United States) to determine cell viability.

### 2.4 Analysis of ROS levels

After treatment with AME and cisplatin for 24 h, the cells were rinsed three times with PBS, and the cells were incubated with 1 μM DCFH-DA, a fluorescent probe, in a light-free environment at 37°C for 30 min to allow complete interaction between the probe and the cellular components. Then, the cells were rinsed 3 times with PBS to remove excess probe. The ROS fluorescence intensity was observed under a fluorescence microscope.

### 2.5 Analysis of lipid peroxidation

The levels of lipid peroxidation in HK-2 cells were analyzed using a Lipid Peroxidation Quantification Assay Kit. HK-2 cells were cultured in confocal Petri dishes (18 × 10^4^/well) and exposed to AME and cisplatin for 24 h. After three consecutive washes with PBS, the cells were treated with 1 μM Liperfluo reagent and incubated in the dark in a cell culture incubator for 30 min. Following three additional PBS washes, the presence of lipid peroxides was quantified and analyzed using a confocal microscope system (Olympus FV3000, Japan).

### 2.6 Animal experiments

Approval for the animal experiments was obtained from the relevant authorities and local committees responsible for regulating animal welfare and use at the First Hospital of Jilin University in Changchun, China. The experiments followed globally accepted standards for the treatment and utilization of laboratory animals. C57BL/6 female mice, aged 8–10 weeks and weighing 18–22 g, were obtained from Liaoning Changsheng Science and Technology Industry Co. Nrf2-targeted knockout mice (Nfe2L2^−/−^) were obtained from Jackson Laboratories, Bar Harbor, United States. The animals were housed at a temperature ranging from 20°C to 23 °C and 40%–80% relative humidity and had free access to food and water. AKI was induced in female C57BL/6 mice using cisplatin after a 1-week acclimation period.

**FIGURE 1:**
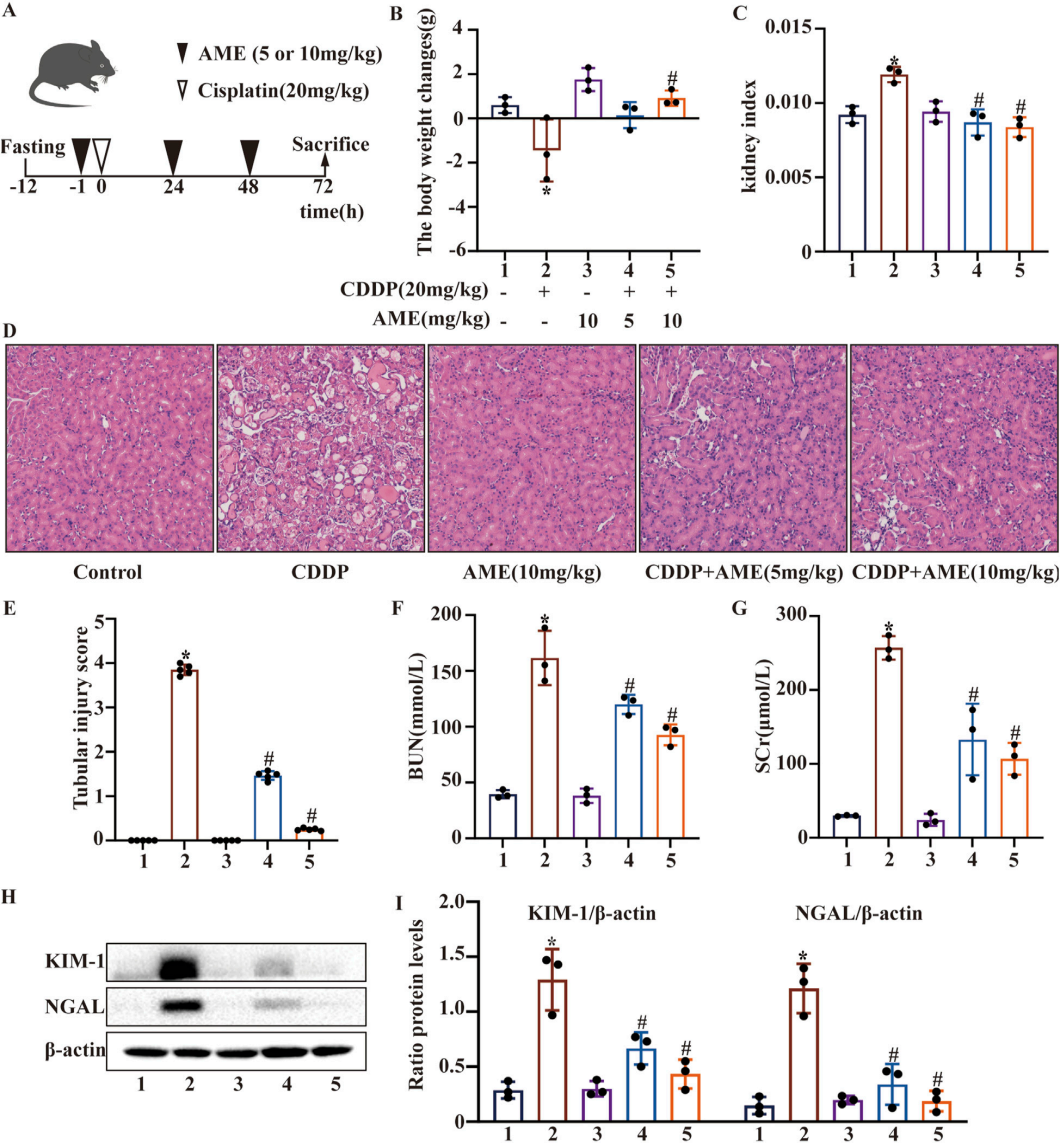
Effects of amentoflavone on cisplatin-induced acute kidney injury **(A)** The experimental design and protocol for cisplatin treatment or combined treatment with amentoflavone involved fasting C57BL/6 mice for 12 h. **(B)** The change in body weight was calculated by subtracting the weight before the initial treatment from the weight before euthanasia. **(C)** The kidney index was calculated as the ratio of kidney weight to weight before euthanasia. **(D, E)** Representative kidney tissue sections were stained with H&E, and kidney injury scores were determined. **(F)** BUN and **(G)** SCr levels were measured. All the data are presented as means ± SEMs (n = 5 per group). **(H, I)** Kidney tissue was homogenized in lysis buffer to extract protein, and Western blotting was performed to assess the protein expression of proximal tubular injury markers, including KIM-1 and NGAL. The experiments were repeated three times. * indicates a significant difference (p < 0.05) between the control group and the CDDP group, and # indicates a significant difference (p < 0.05) between the CDDP group and the AME treatment groups. NS, not significant.

Mice were divided into five groups, with five mice in each group: control group, cisplatin group (20 mg/kg), AME group (10 mg/kg), low-dose treatment group (cisplatin 20 mg/kg + AME 5 mg/kg) and high-dose treatment group (cisplatin 20 mg/kg + AME 10 mg/kg). The experimental protocol is shown in Figure 1A. A second group of mice was divided into two groups, with 15 mice in each group: wild-type group and Nrf2 knockout group. The Nrf2 knockout group consisted of three groups: control group, cisplatin group (20 mg/kg), and treatment group (20 mg/kg cisplatin +10 mg/kg AME).

### 2.7 Histopathologic assessment

Kidney tissue sections were prepared by immersion in a 4% paraformaldehyde solution overnight. The tissue was then embedded in paraffin, sliced thinly, and stained with hematoxylin-eosin (HE). Finally, the sections were viewed under an Olympus Japan light microscope. Kidney damage severity was graded by independent observers who carefully examined five randomly selected areas in each sample. Renal tissue damage was graded as follows: 0, no damage (0%–10%); 1, mild damage (11%–25%); 2, moderate damage (26%–50%); 3, severe damage (51%–75%) and 4, very severe damage (76%–100%). For each section, we randomly selected at least five different renal regions for evaluation.

### 2.8 Measurement of kidney function indicators and quantification of GSH, MDA, MPO, and SOD concentrations

Renal function was evaluated through the quantification of blood serum levels of urea nitrogen and creatinine. Additionally, the concentrations of GSH, MDA, MPO, and SOD were measured in kidney tissue and cell homogenates using kits and protocols provided by the manufacturer.

### 2.9 Iron content analysis

HK-2 cells were seeded in confocal dishes and placed in a 5% CO_2_ cell culture incubator at 37°C overnight, after which the cells were treated with AME and cisplatin for 24 h to observe changes in intracellular Fe^2+^. The cells were then incubated with FerroOrange staining solution (1 μM) (Dojindo; L374, Kumamoto, Kyushu, Japan) at 37°C in a 5% CO_2_ incubator. Cell fluorescence was captured using a laser scanning confocal microscope. An Iron Assay Kit (catalog no. I291) was used to measure serum iron levels in mice.

### 2.10 RNA interference

HK-2 cells were transfected with si-Nrf2(Hanbio Biotechnology Co., Ltd.) according to the manufacturer’s instructions. The interference efficiency was then assessed by Western blotting using an antibody against Nrf2, and the siRNA with the highest interference efficiency was used for subsequent experiments. The siRNA sequences are shown in Table 1.

**TABLE 1:** Sequences of siRNAs for RNA interference.

	Sense sequence	Antisense sequence
Si-Nrf2	CCG​GCA​UUU​CAC​UAA​ACA​CAA​TT	UUG​UGU​UUA​GUG​AAA​UGC​CGG​TT

### 2.11 Western blot analysis

Cells and kidney tissue samples were collected and lysed in cell lysis solution for 30 min on ice. The protein content was measured using a BCA protein assay kit. For kidney tissue and cells, 40 μg and 10 μg, respectively, was resolved by 10%–15% SDS‒PAGE. The proteins were transferred to PVDF membranes. They were then incubated in PBS supplemented with 5% skim milk at ambient temperature for 1 h. Next, the membranes were incubated with primary antibodies overnight at 4°C. Finally, the membranes were incubated at ambient temperature with the appropriate secondary antibodies. Protein bands were detected through the use of enhanced chemiluminescence (ECL). The intensity of the protein bands was measured with ImageJ software.

### 2.12 Statistical analysis

The data are presented as the mean and standard error of the mean (SEM) and were analyzed in GraphPad Prism 10(GraphPad Prism Inc., United States) statistical software. To compare experimental data between groups, one-way analysis of variance (ANOVA) was used. A p value less than 0.05 was considered to indicate statistical significance.

## 3 Results

### 3.1 Effects of AME on CI-AKI

After fasting for 12 h, the mice were administered cisplatin (20 mg/kg) via a single intraperitoneal injection to induce AKI (Figure 1A). We monitored and analyzed changes in the body weight of the mice, and the results indicated a notable reduction in the weight of mice in the model group. Additionally, the body weight of mice was higher in the treatment group than in the model group. Treatment with 10 mg/kg AME resulted in a more pronounced change in mouse body weight (Figure 1B). Based on the kidney index results, AME diminished the kidney damage caused by cisplatin (Figure 1C). To elucidate the mechanism of action of AME, we conducted HE staining and scored the stained sections. Compared with the control group, the cisplatin group exhibited more tissue injury, including necrosis, cast formation, brush border loss, tubular degeneration, and vacuolization. Notably, these tissue anomalies were significantly reduced in the AME-treated mice (Figures 1D,E). Additionally, AME significantly reversed the elevated levels of BUN and SCr noted after 72 h of treatment with cisplatin (Figures 1F,G). Furthermore, the marked increase in the levels of KIM-1 and NGAL induced by cisplatin was attenuated (Figures 1H,I).

### 3.2 AME ameliorated cisplatin-induced ferroptosis and PANoptosis in mice

**FIGURE 2:**
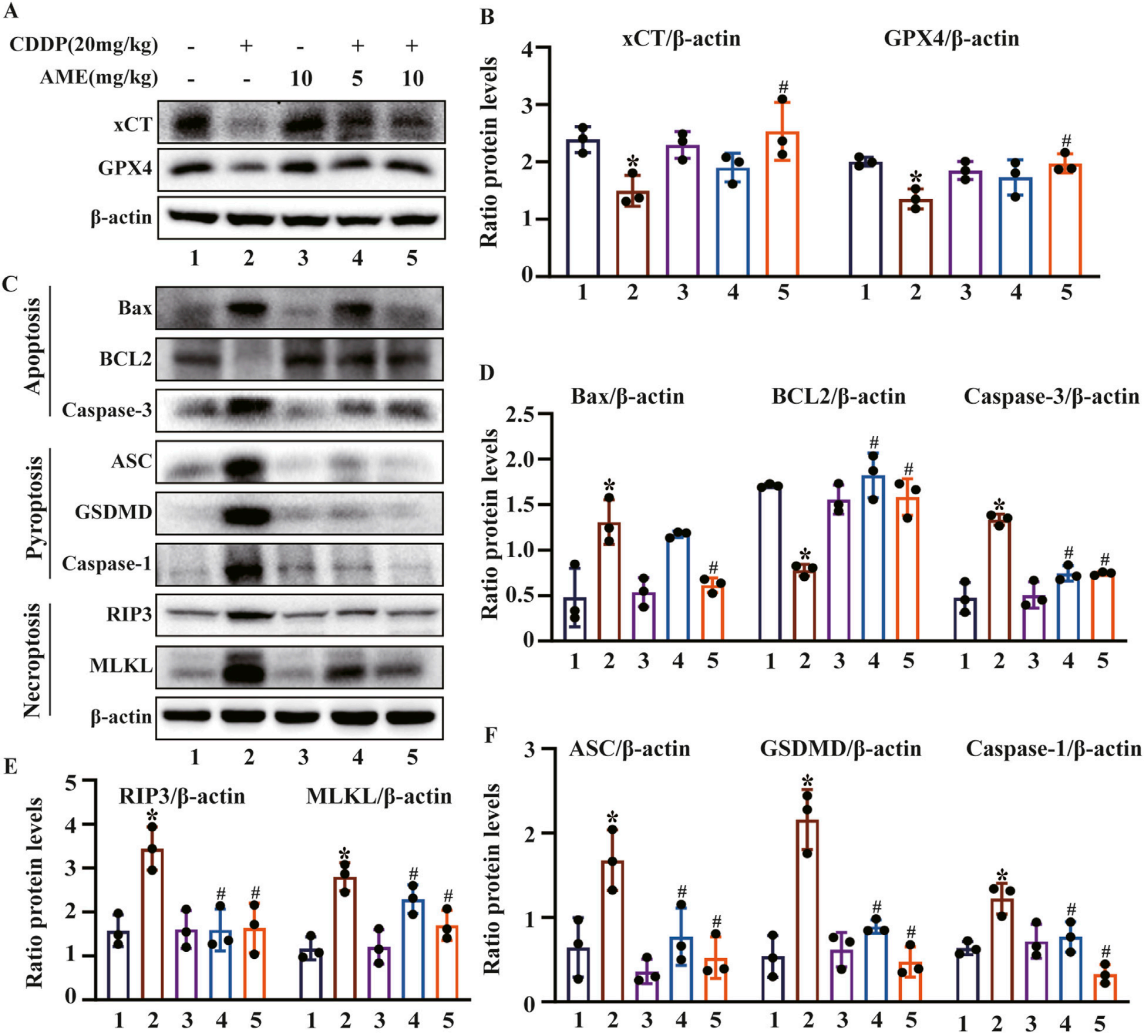
Effects of amentoflavone on cisplatin-induced ferroptosis and the PANoptosis pathway *in vivo* **(A–B)** The expression of xCT and GPX4 in kidney tissue was determined using immunoblotting. **(C–F)** The levels of proteins related to apoptosis (BCL2, Bax, and Caspase-3), pyroptosis (GSDMD, ASC, and Caspase-1), and necroptosis (RIP3 and MLKL) were measured. The experiments were repeated three times. * indicates a significant difference (p < 0.05) between the control group and the CDDP group, and # indicates a significant difference (p < 0.05) between the CDDP group and the AME treatment groups. NS, not significant.

We investigated the effects of AME on the ferroptosis defense proteins xCT and GPX4 in the kidney. The expression levels of xCT and GPX4 were significantly reduced by cisplatin, which was suppressed by AME treatment (Figures 2A,B). PANoptosis is an emerging recognized PCD pattern characterized by apoptosis, pyroptosis and necroptosis. We investigated the occurrence of PANoptosis in CI-AKI and the effect of AME treatment on PANoptosis. In the cisplatin-treated model group, the expression of BCL2 (an apoptosis marker) was decreased, while the expression of Bax and Caspase-3 (apoptosis markers), GSDMD, ASC, and Caspase-1 (pyroptosis markers), and RIP3 and MLKL (necroptosis markers) were increased. AME treatment causes these protein levels to recover (Figures 2C–F).

### 3.3 AME reduces cisplatin-induced redox imbalance

**FIGURE 3:**
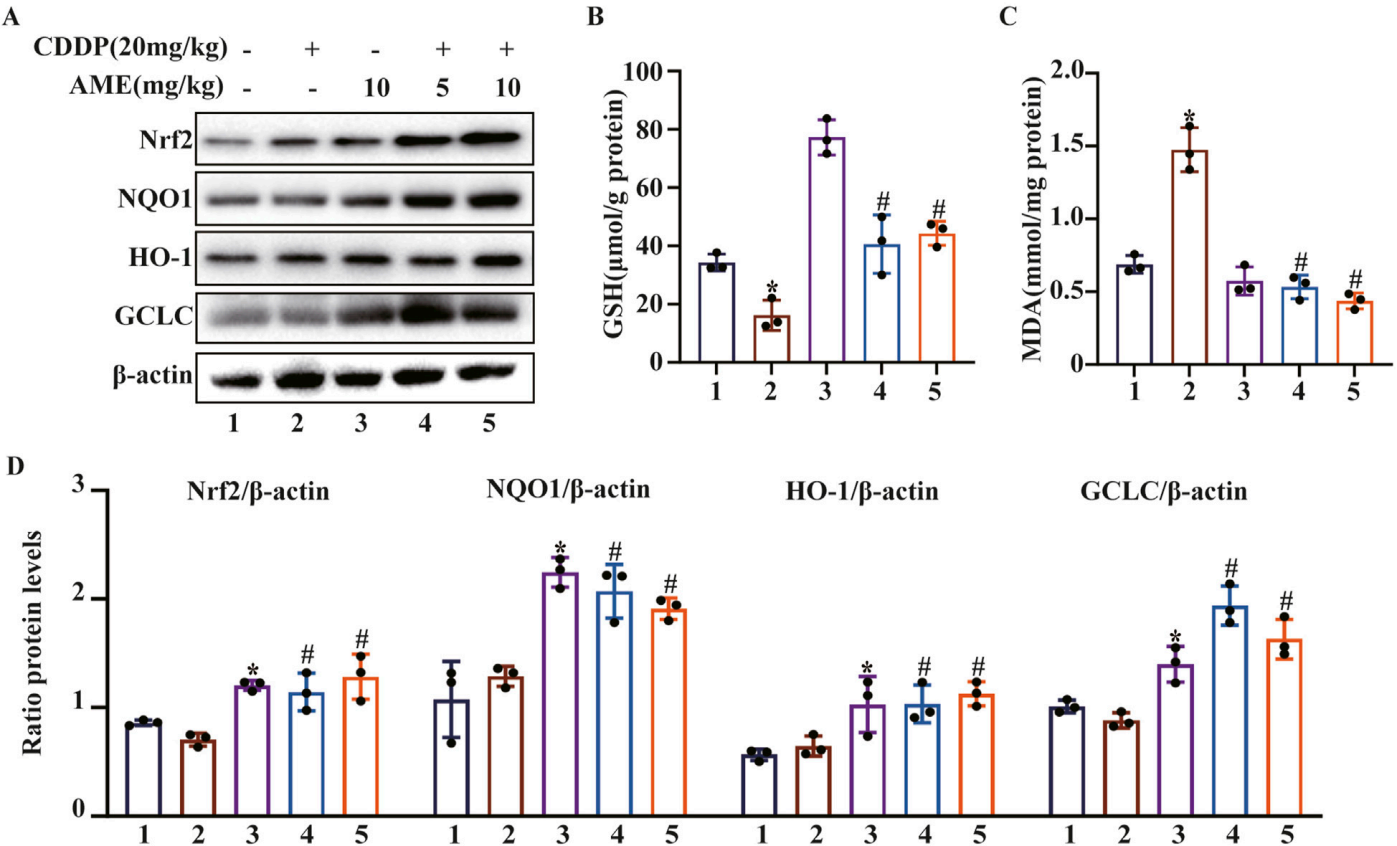
Effects of amentoflavone on oxidative pathway-related proteins in the kidney tissues of cisplatin-treated mice. **(A, D)** Representative Western blots demonstrating the impact of amentoflavone treatment on the levels of oxidative pathway-related proteins, including Nrf2, HO-1, NQO1, and GCLC, in mouse kidney tissues. **(B–C)** The production of GSH and MDA in renal tissues was measured in the mice. The experiments were repeated three times. * indicates a significant difference (p < 0.05) between the control group and the CDDP group, and # indicates a significant difference (p < 0.05) between the CDDP group and the AME treatment groups. NS, not significant.

To evaluate the severity of cisplatin-induced redox imbalance, we quantified markers of oxidative stress by extracting proteins from renal tissues and performing Western blotting. The results confirmed that AME increased Nrf2 expression and increased the levels of the downstream proteins HO-1, NQO1, and GCLC (Figures 3A,D). Additionally, cisplatin was found to reduce GSH concentrations while increasing MDA levels. Consistently, compared to cisplatin treatment alone, AME reduced CI-AKI in mice by increasing GSH levels and reducing MDA levels (Figures 3B,C).

### 3.4 AME provides cellular protection by activating the expression of Nrf2

**FIGURE 4:**
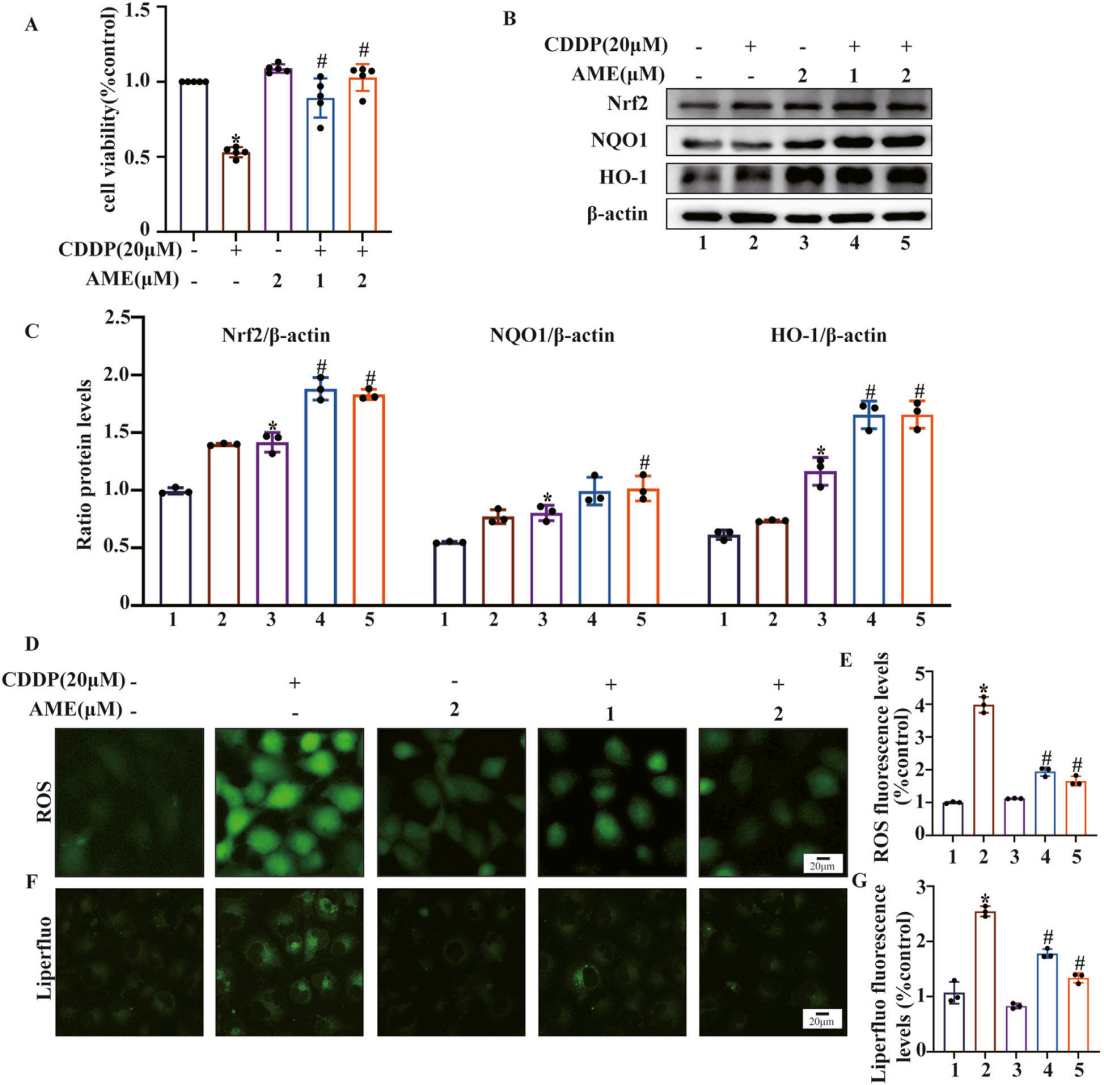
Amentoflavone reduces oxidative stress and lipid peroxidation induced by cisplatin in human kidney 2 (HK-2) cells. **(A)** HK-2 cells were treated with different concentrations of amentoflavone (1 or 2 μM) for 1 h and then with cisplatin (20 μM) for 24 h. Cell viability was assessed using the CCK-8 assay. **(B, C)** The levels of oxidative pathway-related proteins, including Nrf2, HO-1, and NQO1, in HK-2 cells were evaluated using Western blot analysis to determine the effect of amentoflavone. **(D)** HK-2 cells were stained with an ROS fluorescent probe (1 μM) for 30 min, and the resulting fluorescence was detected using a fluorescence microscope. **(E)** ROS fluorescence quantification analysis. **(F)** Liperfluo staining and confocal microscopy were used to assess lipid peroxidation in HK-2 cells. **(G)** Liperfluo fluorescence quantification analysis. * indicates a significant difference (p < 0.05) between the control group and the CDDP group, and # indicates a significant difference (p < 0.05) between the CDDP group and the AME treatment groups. NS, not significant.

This study initially employed the CCK8 assay to evaluate the harmful effects of cisplatin on cells and the shielding effects of AME. Cell death induced by cisplatin (20 μM) was inhibited by AME, leading to a concentration-dependent increase in cell viability (Figure 4A). AME activated Nrf2, NQO1 and HO-1 expression, which protected HK-2 cells from oxidative stress caused by cisplatin, as shown in Figures 4B,C. Reactive oxygen species (ROS) are a class of oxidative molecules. Excessive production of ROS in cells, beyond the clearance capacity of the cells, can lead to oxidative stress. Figure 4D and E shows that cisplatin treatment increased the generation of ROS in HK-2 cells. However, AME inhibited the increase in ROS and exerted a protective effect on the cells. Furthermore, lipid peroxidation increased in response to cisplatin treatment but was reduced by AME, as detected by the Liperfluo probe (Figures 4F,G).

### 3.5 AME inhibits cisplatin-induced ferroptosis and PANoptosis in HK-2 cells

**FIGURE 5:**
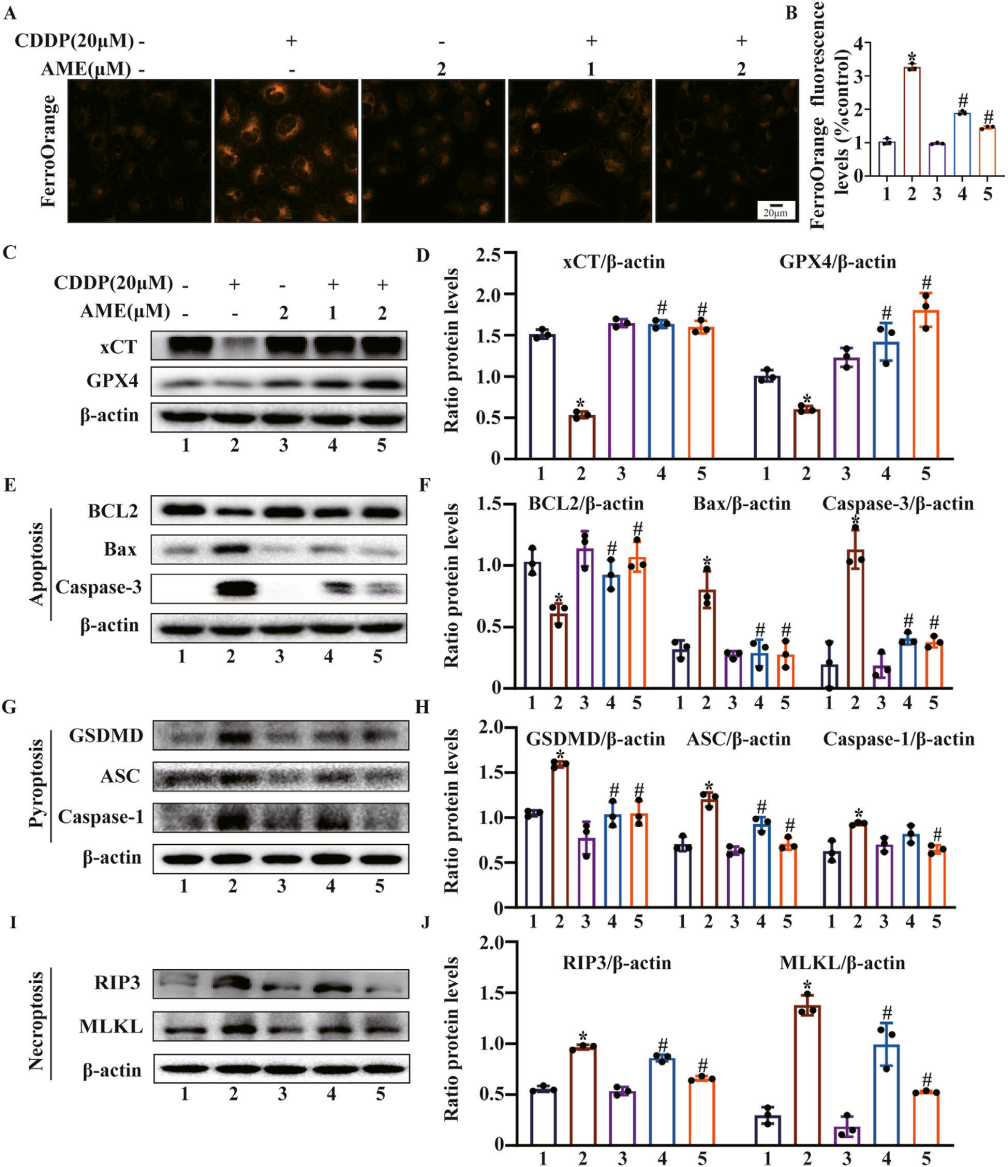
Amentoflavone inhibits ferroptosis and PANoptosis in HK-2 cells. **(A, B)** The iron content in HK-2 cells was assessed by FerroOrange staining. **(C, D)** The protein levels of xCT and GPX4. **(E, F)** The levels of proteins related to apoptosis (BCL2, Bax, and Caspase-3). **(G, H)** The levels of proteins involved in pyroptosis (GSDMD, ASC, and Caspase-1). **(I, J)** The levels of proteins related to necroptosis (RIP3 and MLKL). The experiments were repeated three times. * indicates a significant difference (p < 0.05) between the control group and the CDDP group, and # indicates a significant difference (p < 0.05) between the CDDP group and the AME treatment groups. NS, not significant.

FerroOrange was used to assess Fe^2+^ levels in HK-2 cells. The cisplatin-treated model group exhibited elevated Fe^2+^ content, while AME treatment resulted in a reduction in Fe^2+^ levels (Figures 5A,B). Furthermore, the levels of the ferroptosis defense proteins xCT and GPX4 were assessed. The expression of xCT and GPX4 was inhibited by cisplatin treatment, while AME treatment increased the expression levels of xCT and GPX4 (Figures 5C,D). We also found that AME treatment reversed the expression of PANoptosis-related proteins induced by cisplatin in both HK-2 cells and mice (Figures 5E–J).

### 3.6 AME exerts antioxidative effects on HK-2 cells through an Nrf2-dependent mechanism

**FIGURE 6:**
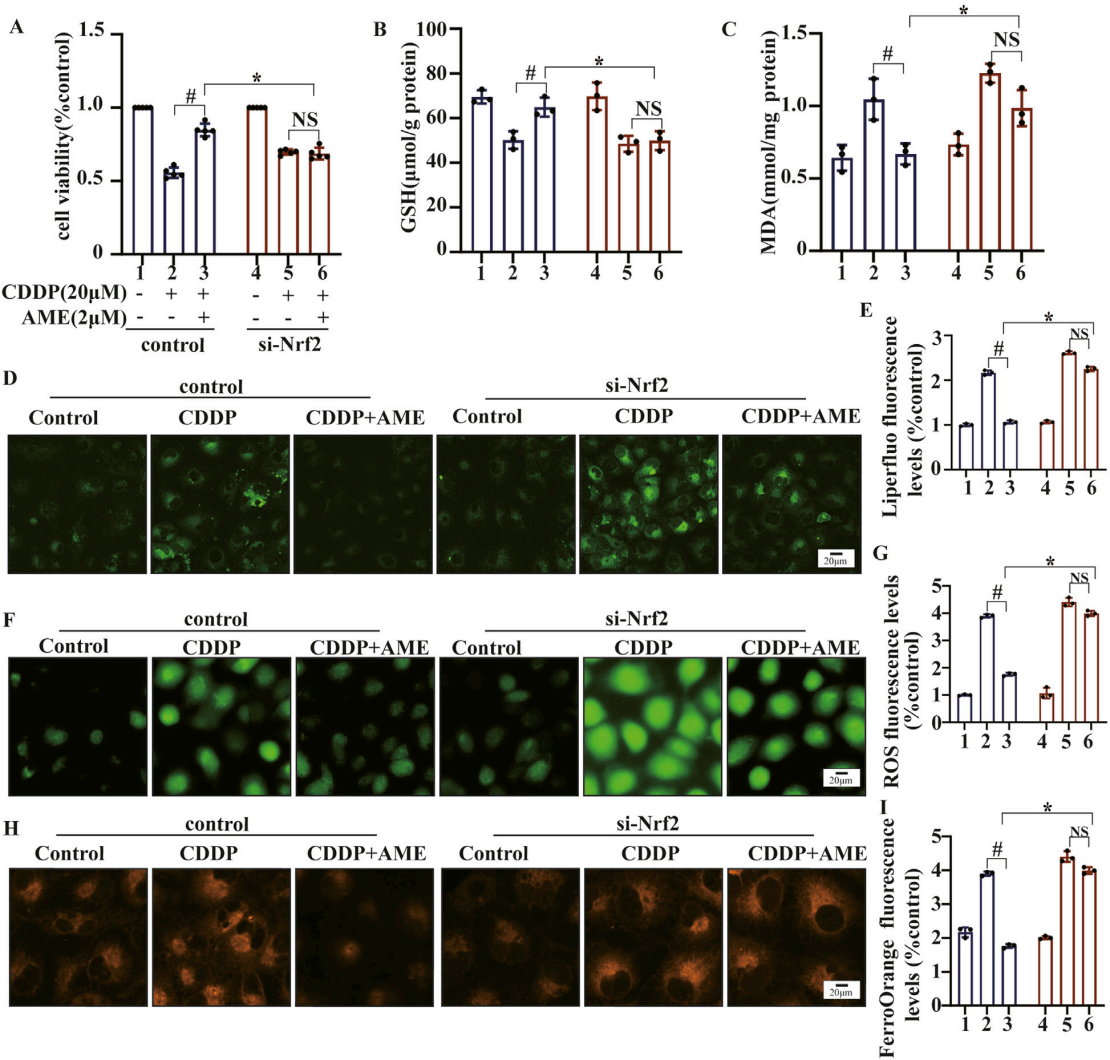
The impact of amentoflavone on oxidative stress and lipid peroxidation in HK-2 cells was found to be dependent on the expression of the Nrf2 gene. **(A)** A Cell Counting Kit-8 (CCK-8) assay was performed to assess the survival rate of the cells. The levels of **(B)** GSH and **(C)** MDA in HK-2 cells were measured using appropriate kits. **(D)** Lipid peroxidation in HK-2 cells was assessed through Liperfluo staining and confocal microscopy. **(E)** LiperFluo fluorescence quantification analysis. **(F)** ROS levels in HK-2 cells were measured using DCFH-DA. **(G)** ROS fluorescence quantification analysis. **(H)** The iron content in HK-2 cells was assessed through FerroOrange staining. **(I)** FerroOrange fluorescence quantification analysis. The experiments were repeated three times. WT indicates normal HK-2 cells, and si-Nrf2 indicates Nrf2 knockdown cells. # indicates a significant difference (p < 0.05) between the CDDP group and the AME treatment groups, and * indicates a significant difference (p < 0.05) between Nrf2 WT and KO mice after AME administration. NS, not significant.

AME did not have any protective effects on Nrf2-knockdown cells (Figure 6A). Moreover, the restorative effects of AME on cisplatin-induced changes in GSH and MDA levels were nullified in Nrf2-knockdown cells, as shown in Figures 6B,C. Similarly, in Nrf2 knockdown cells, the normalizing influence of AME on ROS levels, lipid peroxidation and Fe^2+^ accumulation was also lost, as depicted in Figures 6D–I.

### 3.7 *In vivo*, Nrf2 deficiency exacerbates CI-AKI

**FIGURE 7:**
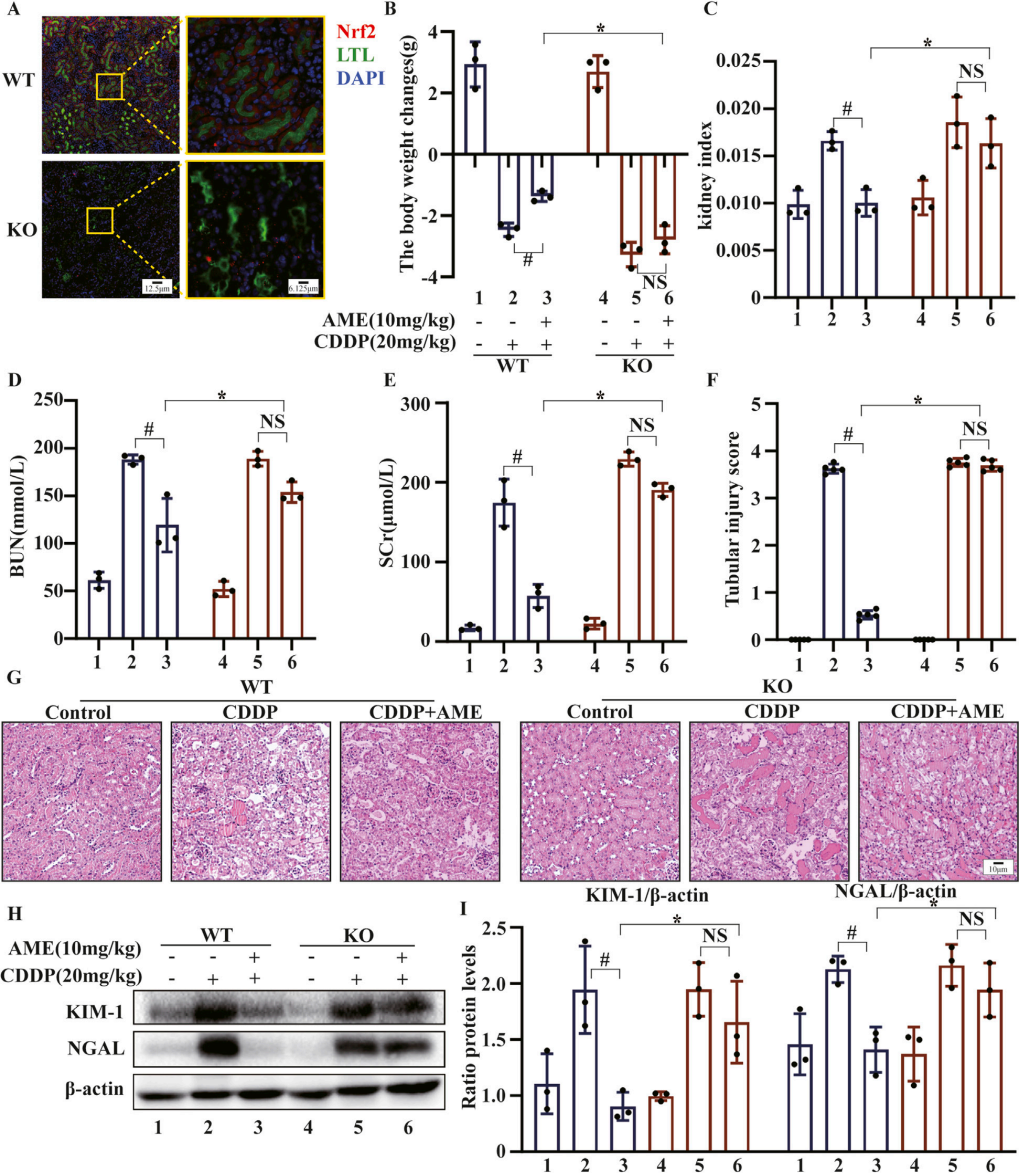
The impact of amentoflavone on cisplatin-induced AKI is contingent on Nrf2 *in vivo*. **(A)** The level of Nrf2 expression in renal tissue was assessed detected using immunofluorescence (IF) staining. Nrf2 is labeled red, LTL is labeled green, and nuclei are labeled blue (DAPI). **(B)** Body weight change was calculated by subtracting the weight before the first treatment from the weight before euthanasia. **(C)** The kidney index was calculated as the kidney weight divided by the weight before euthanasia. **(D)** BUN and **(E)** SCr levels were measured. **(F, G)** Kidney injury scores were calculated, and representative kidney tissue sections were stained with H&E. **(H, I)** Proximal tubular injury markers, including KIM-1 and NGAL, were assessed using Western blotting to determine protein expression. The experiments were repeated three times. WT indicates wild-type mice, and KO indicates Nrf2 knockout mice. # indicates a significant difference (p < 0.05) between the CDDP group and the AME treatment groups, and * indicates a significant difference (p < 0.05) between Nrf2 WT and KO mice after AME administration. NS, not significant.

To investigate whether Nrf2 enhances the protective effects of AME against CI-AKI, we used a murine model to induce AKI in both wild-type and Nrf2-knockout C57BL/6 mice. As demonstrated in Figure 7A, Nrf2 was not expressed around the renal tubules (LTL, green) of Nrf2 (red)-knockout mice. The therapeutic potential of AME was assessed based on several parameters, including body weight (Figure 7B), kidney index (Figure 7C), BUN (Figure 7D), and SCr (Figure 7E). Notably, in Nrf2^−/−^ mice, the therapeutic efficacy of AME was nullified across all these parameters. Histological analysis using HE staining confirmed the loss of the protective effect of AME against CI-AKI. This was evidenced by morphological damage, such as necrosis, cast formation, disruption of brush borders, tubular degeneration, and vacuolization, observed in the Nrf2 knockout group, which resembled that in the model group (Figures 7F,G). Furthermore, the evaluation of the expression of the proximal tubular injury markers KIM-1 and NGAL revealed that the Nrf2^−/−^ mice treated with AME had levels of KIM-1 and NGAL expression similar to those in the model group (Figures 7H,I).

### 3.8 AME alleviates CI-AKI through Nrf2-dependent ferroptosis and partially through Nrf2-dependent PANoptosis

**FIGURE 8:**
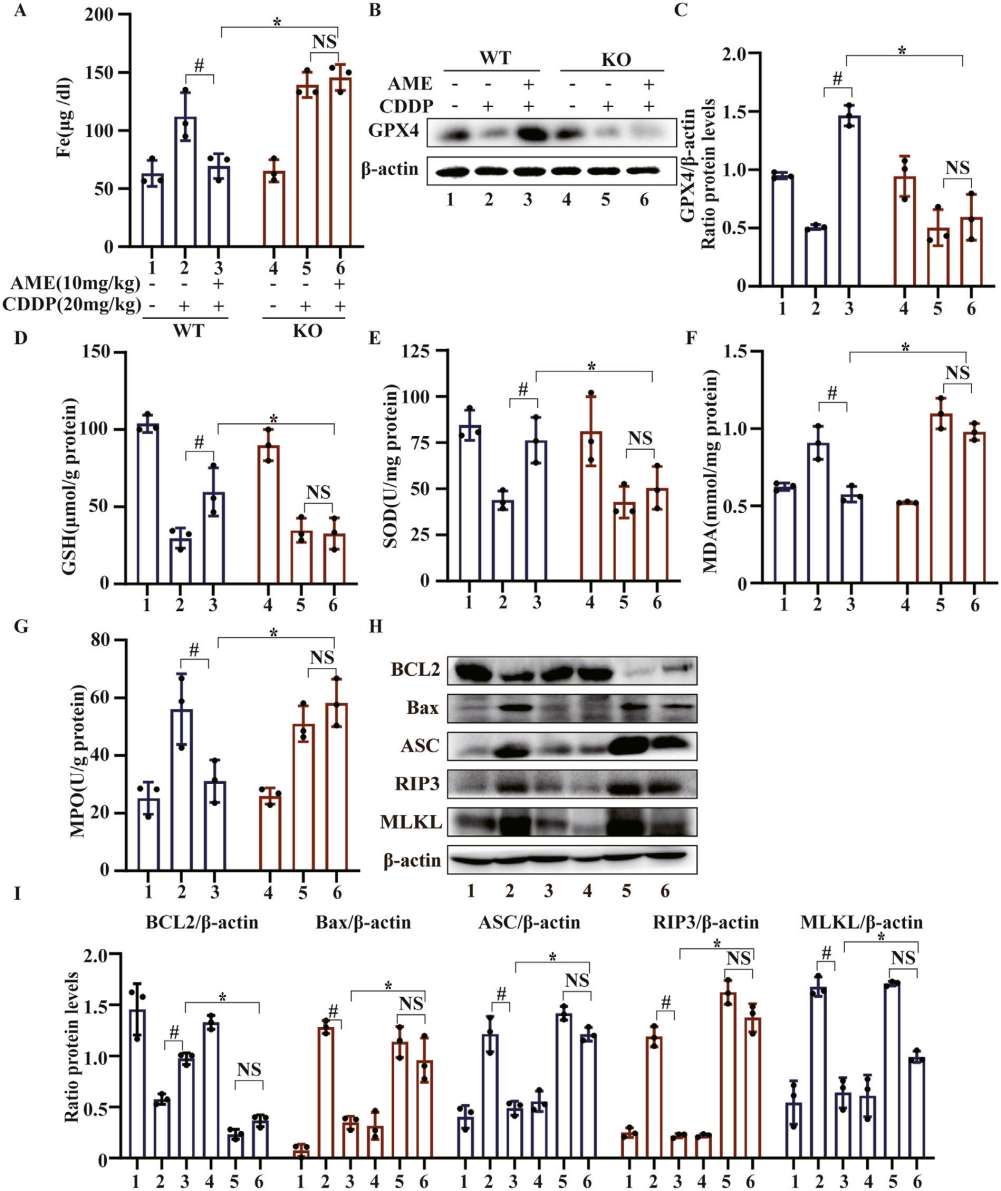
The effects of amentoflavone occur through Nrf2-dependent ferroptosis and partially through Nrf2-dependent PANoptosis. **(A)** Iron metabolism in mice was assessed by measuring the serum iron ion content. **(B,C)** Western blotting was used to assess the protein expression level of GPX4 in renal tissues. Renal tissues were collected from the mice to measure **(D)** GSH, **(E)** SOD, **(F)** MDA, and **(G)** MPO levels. **(H, I)** The protein levels of BCL2, Bax, ASC, and MLKL were assessed in renal tissue via Western blot analysis. The experiments were repeated three times. WT indicates wild-type mice, and KO indicates Nrf2 knockout mice. # indicates a significant difference (p < 0.05) between the CDDP group and the AME treatment groups, and * indicates a significant difference (p < 0.05) between Nrf2 WT and KO mice after AME administration.

In mice, AME treatment led to a reduction in serum iron levels that increased after treatment with cisplatin. However, in Nrf2^−/−^ mice, AME treatment did not affect the serum iron concentration, which was similar to that in the model group (Figure 8A). We also evaluated the expression of GPX4 in WT and Nrf2^−/−^ mice. The results showed that the restorative effect of AME on cisplatin-induced GPX4 reduction was nullified in Nrf2^−/−^ mice (Figures 8B,C). Moreover, in Nrf2^−/−^ mice, the protective effects of AME against the depletion of GSH and SOD, as well as the generation of MDA and MPO, were significantly reduced (Figures 8D–G). Previously, we demonstrated that AME mitigates CI-AKI by upregulating Nrf2; furthermore, the therapeutic effect of AME on ferroptosis in CI-AKI was nullified in Nrf2^−/−^ mice. Changes in the PANoptosis-related proteins BCL2, Bax, ASC, RIP3, and MLKL were measured in WT and Nrf2^−/−^ mice treated with AME for the treatment of cisplatin-induced acute kidney injury. The results indicated that the therapeutic effect of AME was diminished in Nrf2^−/−^ mice but not completely eliminated (Figures 8H,I). The difference between the Nrf2 knockout treatment group and the model group was not statistically significant, suggesting that factors other than Nrf2 potentially influence the effect of AME on CI-AKI-associated PANoptosis.

## 4 Discussion

Cisplatin is one of the most common drugs that causes acute kidney injury (AKI). To date, there remains an unmet need for effective prevention and treatment strategies for CI-AKI. Natural products are viewed as potential agents for treating CI-AKI because of their wide range of biological activities. The results of our study indicated that AME, a natural product with anti-inflammatory and antioxidant properties, can prevent CI-AKI, providing a potential therapeutic agent for the treatment of AKI.

Renal tubule cells are susceptible to a range of toxic and metabolic damage that can lead to cell death. Research has shown that drugs can alleviate cisplatin-induced renal tubular cell apoptosis by reducing the levels of cleaved Caspase-3 and cleaved PARP, as well as the Bax/Bcl-2 ratio (Zhang J. et al., 2023); inhibiting NF-κB-mediated activation of the NLRP3/Caspase-1/GSDMD pathway to alleviate cisplatin-induced renal tubular cell pyroptosis (Jiang et al., 2021); and inhibiting RIP1/RIP3/MLKL-mediated programmed necroptosis (Wu et al., 2020), thereby suppressing CI-AKI. In our research, AME ameliorated cisplatin-induced renal impairment and histopathological damage in mice. Additionally, AME effectively reduced the expression of Bax and Caspase-3, which are proapoptotic proteins, while simultaneously promoting the expression of the antiapoptotic protein BCL2. Similarly, AME inhibited the expression of GSDMD, ASC, and Caspase-1, which mediate pyroptosis, and RIP3 and MLKL, which mediate necroptosis, suggesting that AME can relieve CI-AKI by inhibiting apoptosis, pyroptosis, and necroptosis, i.e., PANoptosis (Zhu et al., 2023). Moreover, our investigation of the mechanisms underlying the protective effects of AME revealed its impact on ferroptosis (Tang et al., 2021), another key pathway implicated in CI-AKI. The results of previous research suggest the involvement of ferroptosis in CI-AKI (Hu et al., 2020; Tang et al., 2021; Kim et al., 2022). Our research revealed that AME alleviated cisplatin-mediated increases in ROS, lipid peroxidation, and iron accumulation in renal tubular epithelial cells. GPX4, an antioxidant defense enzyme, serves as an essential controller of ferroptosis in various cell types (Bersuker et al., 2019). A lack of GPX4 induces ferroptosis, leading to acute renal failure in mice. Additionally, the cystine/glutamate antiporter SLC7A11/xCT is the primary source of cysteine for GSH (Koppula et al., 2018). The inhibition of xCT or a reduction in GPX4 has been shown to ultimately result in oxidative damage and ferroptosis. AME restored the levels of the ferroptosis-related proteins xCT and GPX4 in cisplatin-treated renal tubular epithelial cells (Lv et al., 2023). Therefore, AME can alleviate CI-AKI by inhibiting PANoptosis and ferroptosis.

Nrf2 is an antioxidant factor that maintains cellular redox homeostasis and prevents the accumulation of reactive substances through downstream target genes (Dodson et al., 2019). The activation of Nrf2 expression has been shown to protect renal function in CI-AKI models. The results of our study indicated that AME activated Nrf2, NQO1, HO-1, and GCLC expression, accompanied by the accumulation of GSH and consumption of MDA. However, in Nrf2 knockout mice, the healing impact of AME on CI-AKI was abolished, and renal function indicators did not normalize, and morphological damage did not resolve. Therefore, we demonstrated that AME protects against CI-AKI by activating Nrf2 expression. Research indicates that Nrf2 contributes to protection against ferroptosis by controlling the ferritin synthesis/degradation signaling network (Pefanis et al., 2019), which causes the labile iron pool (Anandhan et al., 2023) within Nrf2-deficient mice to contribute to more severe renal damage and increased iron cell death in CI-AKI. Given the inseparable connection between AME activation of Nrf2 expression and ferroptosis, we used Nrf2 knockout mice to investigate the role of AME in ferroptosis associated with CI-AKI. Notably, Nrf2 deficiency attenuated the protective effect of AME against ferroptosis, as indicated by elevated serum iron and decreased GPX4 levels (Figure 8). Similarly, Nrf2 activation alleviates apoptosis in doxorubicin-induced cardiomyopathy (Zhang W. et al., 2023)and suppresses astrocyte necroptosis induced by traumatic brain injury (Zhao et al., 2022), and the inhibition of Nrf2 limits the alleviation of pyroptosis in gallic acid-induced gouty arthritis cells (Lin et al., 2020). Therefore, we investigated whether the role of AME in regulating cisplatin-induced PANoptosis is also mediated by Nrf2. The results showed that the suppressive impact of AME on PANoptosis in Nrf2 knockout mice was not completely abolished but was weakened compared to that in wild-type mice, suggesting that in addition to affecting Nrf2, AME may regulate other factors to exert its regulatory effects on PANoptosis in CI-AKI.

## 5 Conclusion

Ultimately, our study demonstrated the safeguarding influence of AME against CI-AKI. The protective effect of AME may involve Nrf2-dependent ferroptosis and the partial regulation of PANoptosis through Nrf2. Our findings provide favorable evidence for the application of AME in preventing CI-AKI. However, the safety and efficacy of AME in clinical practice need to be further verified. These research findings provide an important theoretical foundation in support of improvements in novel therapeutic approaches and drugs to improve the management of kidney injury induced by cisplatin and other chemotherapy agents, thereby enhancing patient survival and treatment outcomes.

## Data Availability

The datasets presented in this study can be found in online repositories. The names of the repository/repositories and accession number(s) can be found in the article/supplementary material.

## Abbreviations

Nrf2/NFE2L2, Nuclear factor erythroid 2-related factor 2; AME, amentoflavone; CI-AKI, cisplatin-induced acute kidney injury; CDDP, cisplatin; BUN, blood urea nitrogen; SCr, serum creatinine; HK-2, Human Kidney-2; ER, endoplasmic reticulum; PCD, programmed cell death; NQO1, NAD(P)H quinone oxidoreductase; RIP3, receptor-interacting serine-threonine kinase 3; GCLC, glutamate cysteine ligase catalytic subunit; HO-1, heme oxygenase; GSDMD, gasdermin D; KIM-1, Kidney injury molecule 1; NGAL, Neutrophil Gelatinase-Associated Lipocalin; xCT, cystine/glutamate antiporter; GPX4, Glutathione Peroxidase 4; Bax, Bcl2 Associated X Protein; BCL2, B-cell lymphoma-2; ASC, Apoptosis associated speck like protein containing a CARD; MLKL, mixed-lineage kinase domain-like; ROS, Reactive Oxygen Species; DCFH-DA, 2′,7′-Dichlorodihydrofluorescein diacetate; MDA, Malondialdehyde; MPO, Myeloperoxidase; GSH, glutathione; SOD, Superoxide Dismutase; PBS, Phosphate Buffered Saline; DMSO, Dimethylsulfoxide.
